# *tert*-Butyl *N*-[(3*R*,4*R*)-1-(2-cyano­acet­yl)-4-methyl­piperidin-3-yl]-*N*-methyl­carbamate

**DOI:** 10.1107/S1600536813013512

**Published:** 2013-05-22

**Authors:** Matthias Gehringer, Michael Forster, Dieter Schollmeyer, Stefan Laufer

**Affiliations:** a Eberhard-Karls-University Tübingen, Auf der Morgenstelle 8, 72076 Tübingen, Germany; b University Mainz, Institut of Organic Chemistry, Duesbergweg 10-14, 55099 Mainz, Germany

## Abstract

The piperidine ring of the title compound, C_15_H_25_N_3_O_3_, adopts a slightly distorted chair conformation with the *cis* substituents displaying an N—C—C—C torsion angle of 43.0 (3)°. The cyano group (plane defined by C—C—C N atoms) is bent slightly out of the plane of the amide group by 13.3 (2)°. The carbamate group is oriented at a dihedral angle of 60.3 (5)° relative to the amide group.

## Related literature

For the biological activity and structure–activity relationships of Tofacitinib {systematic name: 3-[(3*R*,4*R*)-4-methyl-3-[meth­yl(7*H*-pyrrolo­[2,3-*d*]pyrimidin-4-yl)amino]­pip­eridin-1-yl]-3-oxo­propane­nitrile} derivatives, see: Changelian *et al.* (2003 ▶); Flanagan *et al.* (2010 ▶); Zerbini & Lomonte (2012 ▶). For details of the synthesis, see: Babu *et al.* (2010 ▶).
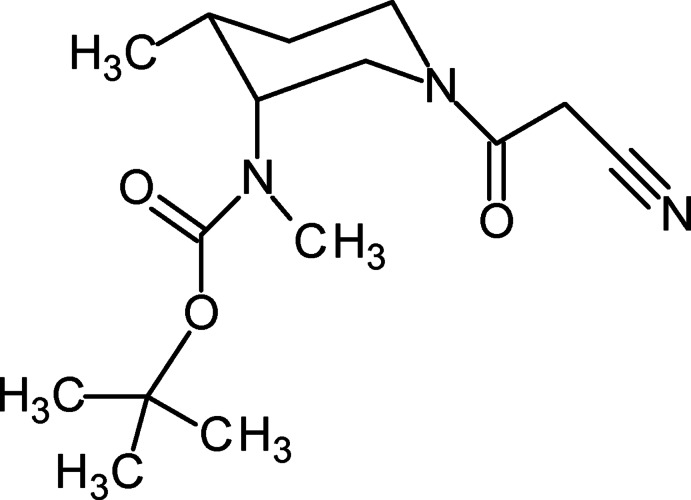


## Experimental

### 

#### Crystal data

C_15_H_25_N_3_O_3_
*M* *_r_* = 295.38
Monoclinic, 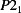

*a* = 7.1786 (11) Å
*b* = 7.3213 (10) Å
*c* = 16.042 (2) Å
β = 102.196 (4)°
*V* = 824.1 (2) Å^3^
*Z* = 2
Mo *K*α radiation
μ = 0.08 mm^−1^
*T* = 173 K
0.60 × 0.35 × 0.10 mm

#### Data collection

Bruker SMART APEXII diffractometer
5061 measured reflections
2115 independent reflections
1818 reflections with *I* > 2σ(*I*)
*R*_int_ = 0.031

#### Refinement

*R*[*F* ^2^ > 2σ(*F* ^2^)] = 0.038
*wR*(*F* ^2^) = 0.101
*S* = 1.03
2115 reflections
195 parameters
1 restraint
H-atom parameters constrained
Δρ_max_ = 0.24 e Å^−3^
Δρ_min_ = −0.17 e Å^−3^

### 

Data collection: *APEX2* (Bruker, 2006 ▶); cell refinement: *SAINT* (Bruker, 2006 ▶); data reduction: *SAINT*; program(s) used to solve structure: *SIR97* (Altomare *et al.*, 1999 ▶); program(s) used to refine structure: *SHELXL97* (Sheldrick, 2008 ▶); molecular graphics: *PLATON* (Spek, 2009 ▶); software used to prepare material for publication: *PLATON*.

## Supplementary Material

Crystal structure: contains datablock(s) I, global. DOI: 10.1107/S1600536813013512/nc2311sup1.cif

Structure factors: contains datablock(s) I. DOI: 10.1107/S1600536813013512/nc2311Isup2.hkl

Supplementary material file. DOI: 10.1107/S1600536813013512/nc2311Isup3.cml

Additional supplementary materials:  crystallographic information; 3D view; checkCIF report

## Figures and Tables

**Fig. 1.**
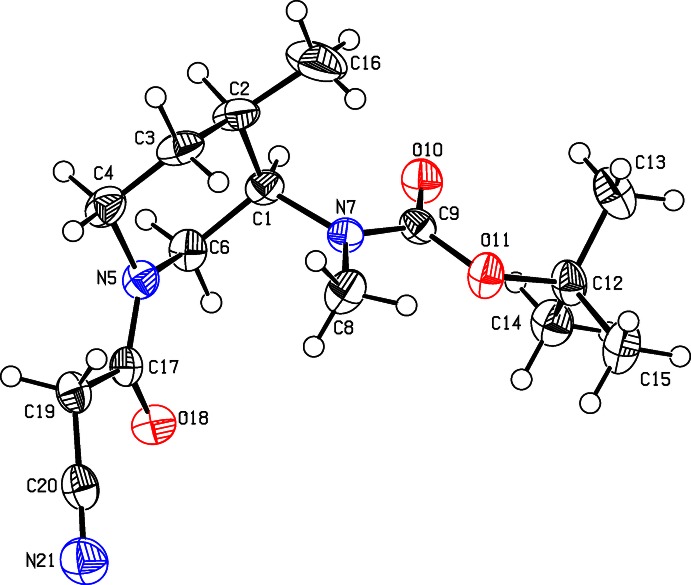
Crystal structure (oder molecular structure) of the title compound with labeling and displacement ellipsoids drawn at the 50% probability level.

## supplementary crystallographic information

### Comment

Tofacitinib (CP690,550) is a potent and selective Janus Kinase (JAK) 1/3 inhibitor (Changelian *et al.* (2003) and Flanagan *et al.*, (2010)) which has recently been approved by the US Food and Drug Administration for the treament of rheumatoid athritis. Furthermore, it is currently in late stage clinical trials for treatment of psoriasis, inflammatory bowel disease, transplant rejection and other immunological disorders (Zerbini & Lomonte, (2012)). Searching for novel JAK inhibitors, the title compound was synthesized as a key intermediate possessing the Boc-protected Tofacitinib side chain.

In the crystal structure of the title compound the piperidine ring adopts a slightly distorted chair conformation with a torsion angle between the *cis* substituents of 43.0 (3)°. The carbamate group shows a dihedral angle of 60.3 (5)° relative to the amide group. The plane defined by atoms C17, C19, C20 and N21 is slightly bent out of the plane of the amide group by 13.3 (2)°.

### Experimental

The title compound was prepared by cyanoacetylation of a precursor possessing a free piperidine NH-function (Babu *et al.*, (2010)) using dicyclohexylcarbodiimide (DCC) and cyanoacetic acid.

*tert*-Butyl-*N*-methyl-*N*-[(3*R*,4*R*)-4-methylpiperidin-3-yl]carbamate (2.80 g, 12.3 mmol) was dissolved in 25 ml of dry methylen chloride and stirred under argon atmosphere. Cyano acetic acid (0.82 g, 9.64 mmol) and *N*,*N*'-dicyclohexylcarbodiimide (1.99 g, 9.64 mmol) was added in one portion while cooling with ice. After 15 min at 273 K, the ice bath was removed, the mixture warmed to room temperature (298 K) and stirring was continued for 3 h. *N*,*N*'-dicyclohexylurea was removed by filtration, washed with methylen chloride and the filtrate concentrated under reduced pressure. Purification by column chromatography (SiO_2_, methylen chloride/ethyl acetate: 7 + 3) yielded the title compound as colorless solid (2.18 g, 84.3%). Crystals of the title compound were obtained by slow evaporation of methanol at room temperature (298 K).

### Refinement

Hydrogen atoms attached to carbons were placed at calculated positions with C—H = 0.95 Å (aromatic) or 0.99–1.00 Å (*sp*^3^ C-atom). All H atoms were refined with isotropic displacement parameters (set at 1.2–1.5 times of the *U*_eq_ of the parent atom). Because no strong anomalous scattering atoms are present Friedel opposites were merged in the refinement. The absolute configuration was assigned according to the synthesis.

### Crystal data

**Table:**

C_15_H_25_N_3_O_3_	*F*(000) = 320
*M**_r_* = 295.38	*D*_x_ = 1.190 Mg m^−^^3^
Monoclinic, *P*2_1_	Mo *K*α radiation, λ = 0.71073 Å
Hall symbol: P 2yb	Cell parameters from 1671 reflections
*a* = 7.1786 (11) Å	θ = 2.6–27.7°
*b* = 7.3213 (10) Å	µ = 0.08 mm^−^^1^
*c* = 16.042 (2) Å	*T* = 173 K
β = 102.196 (4)°	Block, colourless
*V* = 824.1 (2) Å^3^	0.60 × 0.35 × 0.10 mm
*Z* = 2	

### Data collection

**Table:**

Bruker SMART APEXII diffractometer	1818 reflections with *I* > 2σ(*I*)
Radiation source: sealed Tube	*R*_int_ = 0.031
Graphite monochromator	θ_max_ = 27.8°, θ_min_ = 2.6°
CCD scan	*h* = −8→9
5061 measured reflections	*k* = −9→9
2115 independent reflections	*l* = −19→21

### Refinement

**Table:**

Refinement on *F*^2^	Primary atom site location: structure-invariant direct methods
Least-squares matrix: full	Secondary atom site location: difference Fourier map
*R*[*F*^2^ > 2σ(*F*^2^)] = 0.038	Hydrogen site location: inferred from neighbouring sites
*wR*(*F*^2^) = 0.101	H-atom parameters constrained
*S* = 1.03	*w* = 1/[σ^2^(*F*_o_^2^) + (0.0542*P*)^2^ + 0.0849*P*] where *P* = (*F*_o_^2^ + 2*F*_c_^2^)/3
2115 reflections	(Δ/σ)_max_ = 0.001
195 parameters	Δρ_max_ = 0.24 e Å^−^^3^
1 restraint	Δρ_min_ = −0.17 e Å^−^^3^

### Special details

**Table:**

Geometry. All e.s.d.'s (except the e.s.d. in the dihedral angle between two l.s. planes) are estimated using the full covariance matrix. The cell e.s.d.'s are taken into account individually in the estimation of e.s.d.'s in distances, angles and torsion angles; correlations between e.s.d.'s in cell parameters are only used when they are defined by crystal symmetry. An approximate (isotropic) treatment of cell e.s.d.'s is used for estimating e.s.d.'s involving l.s. planes.
Refinement. Refinement of *F*^2^ against ALL reflections. The weighted *R*-factor *wR* and goodness of fit *S* are based on *F*^2^, conventional *R*-factors *R* are based on *F*, with *F* set to zero for negative *F*^2^. The threshold expression of *F*^2^ > σ(*F*^2^) is used only for calculating *R*-factors(gt) *etc*. and is not relevant to the choice of reflections for refinement. *R*-factors based on *F*^2^ are statistically about twice as large as those based on *F*, and *R*- factors based on ALL data will be even larger.

### Fractional atomic coordinates and isotropic or equivalent isotropic displacement parameters (Å^2^)

**Table:**

	*x*	*y*	*z*	*U*_iso_*/*U*_eq_	
C1	0.4456 (3)	0.3404 (3)	0.18701 (13)	0.0302 (4)	
H1	0.4716	0.2110	0.1731	0.036*	
C2	0.6397 (3)	0.4395 (4)	0.20174 (18)	0.0405 (6)	
H2	0.7050	0.3945	0.1564	0.049*	
C3	0.6252 (3)	0.6475 (3)	0.19155 (17)	0.0399 (6)	
H3A	0.5813	0.7008	0.2408	0.048*	
H3B	0.7528	0.6983	0.1911	0.048*	
C4	0.4877 (3)	0.7001 (3)	0.10969 (16)	0.0395 (5)	
H4A	0.4737	0.8346	0.1066	0.047*	
H4B	0.5387	0.6592	0.0601	0.047*	
N5	0.3008 (3)	0.6153 (3)	0.10671 (12)	0.0312 (4)	
C6	0.3066 (3)	0.4151 (3)	0.10847 (14)	0.0330 (5)	
H6A	0.3449	0.3702	0.0563	0.040*	
H6B	0.1772	0.3677	0.1081	0.040*	
N7	0.3602 (2)	0.3322 (2)	0.26269 (11)	0.0285 (4)	
C8	0.2959 (4)	0.4987 (3)	0.29674 (17)	0.0402 (6)	
H8A	0.3926	0.5941	0.2989	0.060*	
H8B	0.2757	0.4757	0.3544	0.060*	
H8C	0.1760	0.5388	0.2599	0.060*	
C9	0.3408 (3)	0.1672 (3)	0.29731 (13)	0.0290 (4)	
O10	0.3892 (2)	0.0216 (2)	0.27102 (10)	0.0394 (4)	
O11	0.2571 (2)	0.1828 (2)	0.36532 (10)	0.0372 (4)	
C12	0.2052 (3)	0.0207 (3)	0.40890 (14)	0.0375 (5)	
C13	0.3833 (4)	−0.0846 (4)	0.45076 (17)	0.0512 (7)	
H13A	0.4434	−0.1362	0.4066	0.077*	
H13B	0.3482	−0.1836	0.4856	0.077*	
H13C	0.4728	−0.0019	0.4870	0.077*	
C14	0.0646 (4)	−0.0948 (4)	0.34738 (17)	0.0433 (6)	
H14A	0.1261	−0.1424	0.3028	0.065*	
H14B	−0.0455	−0.0200	0.3212	0.065*	
H14C	0.0221	−0.1970	0.3782	0.065*	
C15	0.1100 (5)	0.1057 (4)	0.47614 (17)	0.0496 (7)	
H15A	0.2007	0.1864	0.5130	0.074*	
H15B	0.0697	0.0088	0.5106	0.074*	
H15C	−0.0014	0.1765	0.4480	0.074*	
C16	0.7654 (4)	0.3823 (5)	0.2870 (2)	0.0702 (10)	
H16A	0.8933	0.4337	0.2919	0.105*	
H16B	0.7736	0.2487	0.2898	0.105*	
H16C	0.7099	0.4277	0.3338	0.105*	
C17	0.1318 (3)	0.7016 (3)	0.09444 (12)	0.0283 (4)	
O18	−0.0215 (2)	0.6227 (2)	0.08811 (11)	0.0374 (4)	
C19	0.1360 (3)	0.9103 (3)	0.09008 (14)	0.0340 (5)	
H19A	0.2374	0.9573	0.1369	0.041*	
H19B	0.1669	0.9482	0.0353	0.041*	
C20	−0.0465 (4)	0.9893 (3)	0.09718 (15)	0.0372 (5)	
N21	−0.1875 (3)	1.0566 (3)	0.10290 (15)	0.0513 (6)	

### Atomic displacement parameters (Å^2^)

**Table:**

	*U*^11^	*U*^22^	*U*^33^	*U*^12^	*U*^13^	*U*^23^
C1	0.0333 (10)	0.0234 (10)	0.0361 (11)	0.0038 (9)	0.0125 (8)	−0.0038 (9)
C2	0.0278 (11)	0.0393 (14)	0.0574 (15)	0.0051 (10)	0.0160 (10)	−0.0009 (12)
C3	0.0269 (10)	0.0385 (14)	0.0575 (15)	−0.0046 (10)	0.0159 (10)	−0.0048 (11)
C4	0.0391 (11)	0.0325 (12)	0.0511 (14)	−0.0060 (10)	0.0187 (10)	0.0034 (11)
N5	0.0344 (9)	0.0239 (9)	0.0359 (10)	−0.0030 (8)	0.0091 (7)	0.0008 (8)
C6	0.0429 (12)	0.0234 (10)	0.0331 (11)	−0.0014 (9)	0.0090 (9)	−0.0037 (9)
N7	0.0327 (8)	0.0233 (9)	0.0310 (9)	0.0052 (8)	0.0099 (7)	−0.0018 (8)
C8	0.0558 (14)	0.0215 (11)	0.0508 (14)	0.0034 (10)	0.0282 (11)	−0.0025 (10)
C9	0.0314 (9)	0.0258 (10)	0.0289 (10)	0.0031 (9)	0.0038 (8)	−0.0018 (9)
O10	0.0546 (10)	0.0241 (8)	0.0414 (9)	0.0088 (8)	0.0147 (7)	−0.0021 (7)
O11	0.0570 (9)	0.0218 (7)	0.0366 (8)	0.0036 (8)	0.0187 (7)	0.0002 (7)
C12	0.0557 (13)	0.0239 (11)	0.0341 (12)	0.0063 (11)	0.0124 (10)	0.0032 (10)
C13	0.0667 (17)	0.0402 (13)	0.0417 (14)	0.0094 (13)	0.0000 (12)	0.0058 (12)
C14	0.0537 (14)	0.0294 (11)	0.0482 (14)	−0.0010 (11)	0.0138 (11)	−0.0000 (11)
C15	0.0788 (18)	0.0346 (12)	0.0431 (14)	0.0021 (13)	0.0300 (13)	0.0024 (11)
C16	0.0317 (12)	0.070 (2)	0.099 (3)	0.0032 (14)	−0.0084 (14)	0.015 (2)
C17	0.0360 (10)	0.0258 (10)	0.0230 (10)	−0.0026 (9)	0.0059 (8)	−0.0023 (8)
O18	0.0355 (8)	0.0322 (8)	0.0445 (9)	−0.0042 (7)	0.0085 (7)	−0.0065 (7)
C19	0.0398 (12)	0.0286 (11)	0.0321 (11)	−0.0018 (10)	0.0044 (9)	0.0035 (9)
C20	0.0506 (14)	0.0270 (11)	0.0325 (11)	0.0005 (11)	0.0058 (10)	−0.0004 (9)
N21	0.0589 (13)	0.0413 (13)	0.0545 (13)	0.0095 (12)	0.0139 (11)	−0.0015 (11)

### Geometric parameters (Å, º)

**Table:**

C1—N7	1.472 (3)	C9—O11	1.357 (3)
C1—C6	1.533 (3)	O11—C12	1.465 (3)
C1—C2	1.544 (3)	C12—C14	1.512 (4)
C1—H1	1.0000	C12—C13	1.522 (3)
C2—C16	1.529 (4)	C12—C15	1.526 (3)
C2—C3	1.533 (4)	C13—H13A	0.9800
C2—H2	1.0000	C13—H13B	0.9800
C3—C4	1.516 (4)	C13—H13C	0.9800
C3—H3A	0.9900	C14—H14A	0.9800
C3—H3B	0.9900	C14—H14B	0.9800
C4—N5	1.470 (3)	C14—H14C	0.9800
C4—H4A	0.9900	C15—H15A	0.9800
C4—H4B	0.9900	C15—H15B	0.9800
N5—C17	1.345 (3)	C15—H15C	0.9800
N5—C6	1.467 (3)	C16—H16A	0.9800
C6—H6A	0.9900	C16—H16B	0.9800
C6—H6B	0.9900	C16—H16C	0.9800
N7—C9	1.349 (3)	C17—O18	1.228 (3)
N7—C8	1.451 (3)	C17—C19	1.530 (3)
C8—H8A	0.9800	C19—C20	1.459 (3)
C8—H8B	0.9800	C19—H19A	0.9900
C8—H8C	0.9800	C19—H19B	0.9900
C9—O10	1.223 (3)	C20—N21	1.147 (3)
			
N7—C1—C6	112.35 (17)	O10—C9—O11	123.9 (2)
N7—C1—C2	114.38 (18)	N7—C9—O11	110.90 (18)
C6—C1—C2	111.59 (19)	C9—O11—C12	121.04 (17)
N7—C1—H1	105.9	O11—C12—C14	110.10 (18)
C6—C1—H1	105.9	O11—C12—C13	110.3 (2)
C2—C1—H1	105.9	C14—C12—C13	112.8 (2)
C16—C2—C3	112.4 (2)	O11—C12—C15	101.76 (19)
C16—C2—C1	110.6 (2)	C14—C12—C15	110.7 (2)
C3—C2—C1	114.31 (19)	C13—C12—C15	110.8 (2)
C16—C2—H2	106.3	C12—C13—H13A	109.5
C3—C2—H2	106.3	C12—C13—H13B	109.5
C1—C2—H2	106.3	H13A—C13—H13B	109.5
C4—C3—C2	111.2 (2)	C12—C13—H13C	109.5
C4—C3—H3A	109.4	H13A—C13—H13C	109.5
C2—C3—H3A	109.4	H13B—C13—H13C	109.5
C4—C3—H3B	109.4	C12—C14—H14A	109.5
C2—C3—H3B	109.4	C12—C14—H14B	109.5
H3A—C3—H3B	108.0	H14A—C14—H14B	109.5
N5—C4—C3	110.11 (18)	C12—C14—H14C	109.5
N5—C4—H4A	109.6	H14A—C14—H14C	109.5
C3—C4—H4A	109.6	H14B—C14—H14C	109.5
N5—C4—H4B	109.6	C12—C15—H15A	109.5
C3—C4—H4B	109.6	C12—C15—H15B	109.5
H4A—C4—H4B	108.2	H15A—C15—H15B	109.5
C17—N5—C6	119.57 (19)	C12—C15—H15C	109.5
C17—N5—C4	126.53 (19)	H15A—C15—H15C	109.5
C6—N5—C4	113.55 (19)	H15B—C15—H15C	109.5
N5—C6—C1	112.52 (19)	C2—C16—H16A	109.5
N5—C6—H6A	109.1	C2—C16—H16B	109.5
C1—C6—H6A	109.1	H16A—C16—H16B	109.5
N5—C6—H6B	109.1	C2—C16—H16C	109.5
C1—C6—H6B	109.1	H16A—C16—H16C	109.5
H6A—C6—H6B	107.8	H16B—C16—H16C	109.5
C9—N7—C8	121.94 (17)	O18—C17—N5	123.7 (2)
C9—N7—C1	118.25 (17)	O18—C17—C19	119.5 (2)
C8—N7—C1	119.80 (18)	N5—C17—C19	116.7 (2)
N7—C8—H8A	109.5	C20—C19—C17	111.4 (2)
N7—C8—H8B	109.5	C20—C19—H19A	109.3
H8A—C8—H8B	109.5	C17—C19—H19A	109.3
N7—C8—H8C	109.5	C20—C19—H19B	109.3
H8A—C8—H8C	109.5	C17—C19—H19B	109.3
H8B—C8—H8C	109.5	H19A—C19—H19B	108.0
O10—C9—N7	125.24 (18)	N21—C20—C19	177.9 (3)
			
N7—C1—C2—C16	43.0 (3)	C2—C1—N7—C8	67.0 (3)
C6—C1—C2—C16	171.9 (2)	C8—N7—C9—O10	178.8 (2)
N7—C1—C2—C3	−85.0 (3)	C1—N7—C9—O10	0.2 (3)
C6—C1—C2—C3	43.9 (3)	C8—N7—C9—O11	−0.3 (3)
C16—C2—C3—C4	−175.6 (2)	C1—N7—C9—O11	−178.92 (17)
C1—C2—C3—C4	−48.5 (3)	O10—C9—O11—C12	−4.7 (3)
C2—C3—C4—N5	55.2 (3)	N7—C9—O11—C12	174.44 (18)
C3—C4—N5—C17	126.0 (2)	C9—O11—C12—C14	−60.1 (3)
C3—C4—N5—C6	−60.9 (3)	C9—O11—C12—C13	65.0 (3)
C17—N5—C6—C1	−129.3 (2)	C9—O11—C12—C15	−177.46 (19)
C4—N5—C6—C1	57.1 (3)	C6—N5—C17—O18	3.4 (3)
N7—C1—C6—N5	83.0 (2)	C4—N5—C17—O18	176.1 (2)
C2—C1—C6—N5	−47.0 (3)	C6—N5—C17—C19	−177.8 (2)
C6—C1—N7—C9	117.2 (2)	C4—N5—C17—C19	−5.1 (3)
C2—C1—N7—C9	−114.3 (2)	O18—C17—C19—C20	12.5 (3)
C6—C1—N7—C8	−61.5 (3)	N5—C17—C19—C20	−166.36 (18)
